# Amplification of cestode DNA from the peri-anal region of naturally infected foxes by PCR and LAMP: proof of concept for a potential sampling strategy for diagnosing human taeniosis

**DOI:** 10.1007/s00436-021-07271-z

**Published:** 2021-08-31

**Authors:** Gillian Muchaamba, Cristian A. Alvarez Rojas, Peter Deplazes

**Affiliations:** Institute of Parasitology, Vetsuisse and Medical Faculty, University of Zürich, 8057 Zürich, Switzerland

**Keywords:** Cestode, DNA, Peri-anal swabs, DNA extraction, Polymerase chain reaction (PCR), Loop-mediated isothermal amplification (LAMP)

## Abstract

The diagnosis of human taeniosis can be achieved through coproscopy, ELISA or PCR. An important limitation of these methods is the high turnaround time for stool sample collection and preparation, indicating the need for a straightforward sampling strategy. Due to the high metabolic activity and reproductive potential of *Taenia* spp.*,* we hypothesise that parasite DNA (cells and eggs) present in the peri-anal region of the host can be exploited as a target for molecular diagnosis. We evaluated the feasibility of recovering parasite DNA from the peri-anal area of foxes naturally infected with *Taenia* spp. Before necropsy, cotton swabs were rubbed at the peri-anal region of foxes. DNA was extracted using alkaline lysis coupled with a commercial DNA isolation kit (method A) or alkaline lysis alone (method B). DNA was used in the multiplex-PCR assay (previously described and called here swab-PCR) and a novel LAMP assay detecting *Taenia* spp. commonly found in foxes (swab-LAMP). The results of these assays from 105 foxes were compared with the presence of intestinal helminths determined at necropsy and by the sedimentation and counting technique (SCT). The sensitivity of swab-PCR for detecting *Taenia* (*n* = 68) was 89.8% (95% CI, 77.7–96.6) and 89.5% (66.9–98.7) using methods A and B, respectively. The sensitivity of the swab-LAMP assay was 83.7% (70.3–92.7) using method A and 89.5% (66.9–98.7) with method B. We postulate that peri-anal swab sampling followed by simplified DNA extraction and LAMP might be a suitable strategy for surveillance of human taeniosis in resource-limited settings in the future.

**Supplementary Information:**

The online version contains supplementary material available at 10.1007/s00436-021-07271-z.

## Introduction

Taeniosis of humans is a food-borne zoonotic infection resulting from the ingestion of unfrozen, raw or undercooked beef containing viable *Taenia saginata* cysticerci or pork containing *Taenia solium* or *Taenia asiatica* cysticerci. *T. saginata* is distributed worldwide (Eichenberger et al. 2020), whereas *T. asiatica* is restricted to Asia (Eom et al. 2009). Taeniosis usually cause mild intestinal disorders characterised by moderate diarrhoea, abdominal discomfort and pruritus ani (Eom and Rim 1992; Tembo and Craig 2015). Individuals infected with *T. saginata* may notice proglottids on clothes, bedding and in the stool. The major burden of *T. saginata* is in the meat industry which suffers considerable economic losses due to the cost of carcass condemnation at meat inspection (Jansen et al. 2018). *T. solium* is endemic in low-income countries, particularly in Asia, Africa and Latin America, where sanitation provision is inadequate and where free-ranging pigs have access to human faeces (Donadeu et al. 2016). Due to the migration of infected humans from endemic areas, *T. solium* has sporadically been reported in Europe, Australia and the USA (Schantz et al. 1998; Del Brutto and Garcia 2012; Gabriel et al. 2015; Forster et al. 2020). Taeniosis caused by *T. solium* may remain unnoticed by carriers as it is characterised by the passive discharge of proglottids in the stool (Pawlowski and Schultz 1972; Garcia et al. 2003). *Taenia solium* is unique amongst the parasites causing human taeniosis because the metacestode stage can also establish in humans resulting in cysticercosis which is a severe disease (Roberts et al. 1994). The transmission occurs through accidental ingestion of viable eggs shed by *T. solium* carriers and possibly by autoinfection in taeniosis patients through retrograde transport of eggs in the small intestines (reverse-peristalsis) (Sanchetee 1998; Garcia and Del Brutto 2000; Kobayashi et al. 2013). Neurocysticercosis (NCC) results from one or more oncospheres developing in the central nervous system causing epilepsy and various other neurological disease manifestations (Garcia et al. 1991). NCC often remains undiagnosed due to insufficient diagnostic tools in endemic areas, and many infected people remain asymptomatic for a long time (Moyano et al. 2016). On the other hand, NCC is an important cause of epilepsy in endemic countries where symptomatic people may suffer from social stigmatisation and discrimination, which complicates the correct and early diagnosis of this pathology (Maudlin et al. 2009).

The correct estimation of the prevalence of *T. solium* taeniosis (and cysticercosis) infection in humans in endemic areas is difficult to achieve due to the lack of cost-effective, highly sensitive and specific diagnostic methods (Donadeu et al. 2017; Dixon et al. 2020). The accurate diagnosis of taeniosis carriers has been identified by the World Health Organization (WHO) as one of the priorities for implementing control strategies against this parasite (Dixon et al. 2020). Laboratory diagnosis of taeniosis by coprological examination detecting eggs has a low sensitivity and is only genus-specific (Gemmell et al. 1983; Li et al. 2013). Antigen detection in faeces (copro-ELISA) is highly sensitive and can detect prepatent infections; however, most of these tests are not species-specific with cross-reactions among *Taenia* species reported (Allan et al. 1990, 1996; Deplazes et al. 1991). In addition, such tests are only available in specialised laboratories. Different studies have shown that the PCR-based approach (copro-PCR) provides high sensitivity for the diagnostics of *Taenia* spp. carriers; nonetheless, their implementation in field conditions is problematic because it depends on costly equipment and trained staff (Gottstein et al. 1991; Yamasaki et al. 2004). Loop-mediated isothermal amplification (LAMP) facilitates DNA-based diagnostics in field conditions as it does not require a thermocycler (Notomi et al. 2000; Nkouawa et al. 2012). LAMP has been used to diagnose human taeniosis carriers and has proven to be more sensitive than multiplex-PCR (Nkouawa et al. 2009, 2010). However, a significant limitation of DNA-based methods, including LAMP, is the high cost of DNA isolation, usually relying on commercial kits, as well as the relatively inefficient collection of stool samples.

There is evidence of the presence of *Taenia* spp. eggs in the peri-anal region of infected humans and dogs based on the scotch tape method initially applied to diagnose *Enterobius* infections in humans (Graham 1941). This method used in humans showed a higher sensitivity than single coprological examination, detecting 90% of patients with *T. saginata* infection (Mazzotti 1944; Hall et al. 1981; de Kaminsky 1991). The scotch tape method showed a sensitivity of 96.7% in a study including 60 samples from 10 dogs experimentally infected with *Taenia hydatigena* during the patent period (Deplazes and Eckert 1988). This method also detected *Echinococcus granulosus* eggs in naturally infected dogs in Kenya with a sensitivity of 73% (eight of 11 dogs were infected, seven dogs harboured > 5,000 worms) (Craig et al. 1988). *Mesocestoide*s spp. are also known to release mature motile proglottids which may be present around the anus and peri-anal region of infected canids (Saari et al. 2019). Considering this and the inherent limitations of current diagnostic methods for taeniosis, we hypothesised that due to the high reproductive potential and size of large cestodes (*Taenia* spp. and *Mesocestoides* spp.), it should be possible to detect parasitic DNA originating from cells/eggs attached to the peri-anal region of the infected host. To support this concept, we used foxes as a model, evaluating the potential of this sampling technique for future studies in human taeniosis. In this study, we also evaluated the use of the cost-effective alkaline lysis of swabs from the peri-anal region of foxes to be used directly as DNA template in multiplex PCR; referred to as swab-PCR in this publication (Trachsel et al. 2007) and swab-LAMP; a LAMP assay developed in this study and aiming to amplify *Taenia* species commonly found in the fox in Europe.

## Materials and methods

### Foxes

Animals used for necropsy were shot during the official hunting season (February and December 2020, January 2021) by certified hunters in Zürich, Switzerland. Foxes were delivered individually in plastic bags within 6 h after being shot. Necropsies and sample collection were performed in a dedicated laboratory under safety precautions and protective gear approved by the Swiss biosafety authorities (biohazard safety level 2 +).

### Collection of peri-anal swabs and necropsy

Before necropsy, a single cotton swab was rubbed at the peri-anal region of each fox, avoiding contact with faecal material if present. Each swab was placed in a 2-ml centrifuge tube, trimmed to allow closure of the tube and labelled. New gloves were used for collecting every sample to avoid cross-contamination. All tubes were placed in sealable bags, stored at − 80 °C for at least 1 week to inactivate *Echinococcus multilocularis* eggs and then stored at − 20 °C for subsequent analysis.

The small intestine of each fox was dissected longitudinally. The detection of parasites was carried out following the sedimentation and counting technique (SCT) previously described by Eckert et al. (2001). The sediments from SCT were fixed in 95% ethanol. Additionally, all large helminths found in each fox were collected and stored in 95% ethanol in 50 ml Falcon tubes. Sediments and worms were stored at − 80 °C for a week before microscopic examination for biosafety reasons. Helminths found in the small intestines were identified according to their morphology and size (Deplazes et al. 2016). Faeces were collected from the rectum of foxes at necropsy to isolate taeniid eggs by sequential sieving and flotation as previously described (Mathis et al. 1996). DNA isolated from these eggs was used as a template in multiplex PCR (Trachsel et al. 2007) to classify taeniid infection in foxes.

### DNA extraction

Cotton swabs were subjected to two DNA extraction methods in this study. Method A consisted of combining alkaline lysis and the QIAamp DNA kit (Qiagen) as previously described (Štefanić et al. 2004). For method B, the peri-anal swabs were treated with NaOH only as previously described (Bucher et al. 2021). Briefly, each cotton swab was incubated with 300 μl of 0.2 N NaOH at 95 °C for 10 min. The tubes were centrifuged at full speed for 5 min, and 5 μl of the supernatant were diluted with 100 mM Tris–HCl (245 μl) and used directly for swab-PCR and swab-LAMP. To assess the capacity of the methods to extract free DNA from cotton swabs, we spiked swabs in triplicate with ten-fold serial dilution from a starting solution containing 10 ng/μl of *Taenia polyacantha* DNA (measured with NanoDrop) isolated from adult worms. We used *T. polyacantha* DNA as a representative of *Taenia* spp. commonly found in foxes.

### Swab-PCR (multiplex PCR) for detection of *Taenia* spp., *Mesocestoides* spp. (large cestodes) and *E. multilocularis*

As mentioned before, we refer in this study to the multiplex-PCR described by Trachsel et al. (2007) as swab-PCR, which can differentiate cestodes including *Taenia* species (267 bp) from *E. multilocularis* (yielding an amplicon of 395 bp). Amplicons were purified using the MinElute PCR Purification Kit (Hilden, Germany) and sequenced unidirectionally using Sanger technology (Microsynth AG, Balgach, Switzerland). Additionally, we used a LAMP assay developed to detect *E. multilocularis* (Bucher et al. 2021) to compare with our results.

### Swab-LAMP (loop-mediated Isothermal amplification) for large cestodes

The target region for the primer design was the 12S rRNA mitochondrial gene sequence. We chose this gene as it was previously used to design the multiplex-PCR amplifying *Taenia* spp. and other large cestodes DNA (Trachsel et al. 2007). No full mitogenome is available in GenBank for *T*. *polyacantha*. The available sequence for 12S rRNA from *T. polyacantha* (DQ408419) is only 313 bp; to acquire a longer sequence for this parasite to be used in an alignment for designing LAMP primers, we amplified the flanking regions (5’ and 3’) using PCR primers designed based on available conserved sequences for other *Taenia* species (primer sequences can be found in Supplementary material; Table S1). Then we aligned the acquired sequence for the 12S rRNA gene for *T. polyacantha* (MZ414196) with the corresponding sequence of *T. crassiceps* (NC002547) and *Mesocestoides litteratus* (JN088186) using the Geneious Prime software 2020. Five primer sets were designed using Primer Explorer v5 (https://primerexplorer.jp/e/). Primers were validated in silico using primer BLAST (https://www.ncbi.nlm.nih.gov/tools/primer-blast/), and their sequences were modified to include ambiguous bases to amplify *Taenia* spp.

All the primers were tested for analytical sensitivity with serial dilutions of *T. polyacantha* DNA (10 ng to 0.1 fg/μl) and analytical specificity with DNA of *T. crassiceps*, *T. polyacantha*, *T*. *solium*, *T. saginata*, *T. ovis*, *T. hydatigena*, *T. krabbei, Hydatigera taeniaeformis*, *T. multiceps*, and non-*Taenia* species including *E. multilocularis*, *E. granulosus* s.s*, M. litteratus, Dipylidium caninum, Diphyllobothrium latum* and *Toxocara canis* from the collection of the Institute of Parasitology (IPZ), University of Zürich. The primers were optimized by using different amplification temperatures (56 °C to 65 °C), malachite green concentrations (0.004%, 0.006% and 0.008%), and betaine concentrations (0.2 M, 0.4 M and 0.8 M). The primer set amplifying *T. crassiceps, T. polyacantha* and *M. litteratus* with the strongest signal was chosen for this study (see supplementary material; Table S2). Alignment of the sequences and the annealing sites of the selected primers can be seen in the Supplementary material, Figure S1.

The swab-LAMP reaction was performed in a 25 μl volume consisting of 0.006% malachite green, 10 × Isothermal Amplification buffer [20 mM Tris–HCl, 10 mM (NH_4_)_2_SO_4,_ 50 mM KCl, 2 mM MgSO_4,_ 0.1% Tween 20 at pH 8.8], 6 mM MgSO4, 1.4 mM dNTPs (all previous reagents were from New England Biolabs, Ipswich, MA, USA), 0.8 M betaine, 25 × primer mix consisting of 1.6 mM each of FIP and BIP, 0.2 mM each of F3 and B3 and 0.4 mM of FL, 8 U/ml Bst DNA polymerase (New England Biolabs, Ipswich, MA, USA), nuclease-free water and 2 μl of template DNA. All components were mixed on ice, and the reaction mixture was incubated at 60 °C for 1 h, followed by 85 °C for 5 min. The results of the swab-LAMP assay were assessed by a colour change and confirmed by electrophoresis on a 2% (w/v) agarose gel stained with Gel Red and visualised using UV light (Bio-Rad Molecular Imager).

### Molecular detection of *Toxocara* spp.

For *Toxocara* species, primers previously designed to amplify DNA from *Toxocara* spp. were used (Guggisberg et al. 2020) to produce and sequence a 227 bp amplicon.

### Validation of the peri-anal cotton swabs for DNA extraction in field samples

Peri-anal swabs were collected from 105 foxes, including swabs from 78 foxes necropsied in February 2020 treated with method A for DNA extraction and swabs from 27 foxes necropsied in December 2020 and February 2021 treated with method B. The number of foxes used for method A and B varied due to availability of animals during the hunting season.

## Results

### Necropsy and SCT

Large cestodes (*Taenia* spp. and *Mesocestoides* spp.) were found in 87 foxes, while 18 foxes were free of large cestodes. *E. multilocularis* was found in 66 foxes and *T. canis* in 43 animals. A detailed description of the results of necropsy and SCT can be found in the supplementary material (Table S3).

### DNA extraction: spiking of swabs with *Taenia* DNA

After spiking peri-anal swabs with DNA from *T. polyacantha,* it was possible to detect 1 pg/μl using PCR and 0.1 ng/μl using LAMP with method A for DNA extraction. The limit of detection with method B was 10 pg/μl using PCR and 1 ng/μl with LAMP.

### Molecular detection

#### LAMP primer analytical sensitivity, specificity and assay optimisation

In-silico blast analysis showed that the designed LAMP primers could amplify *Taenia* spp. and *Mesocestoides* spp. which was further validated using the swab-LAMP assay amplifying DNA from *T. crassiceps*, *T. polyacantha*, *T. multiceps*, *T. ovis*, *T. saginata*, *T. solium*, *T. hydatigena*, *Hydatigera taeniaeformis* and *M. litteratus,* but not from *T. krabbei*, *E. multilocularis*, *E. granulosus* s.s*, Dipylidium caninum*, *Diphyllobothrium latum* and *T. canis*. The analytical sensitivity assay showed that the limit of detection of the designed LAMP primers was 1 pg/μl. Positive swab-LAMP samples showed a cyan colour change, while the negative samples were colourless. On gel electrophoresis, positive amplicons showed a characteristic ladder-like pattern.

#### Amplification of large cestode DNA in foxes using peri-anal swabs

From the 78 peri-anal swabs using method A for DNA extraction, 49 were positive for *Taenia* spp. at necropsy/SCT, and 52 were positive for *Mesocestoides* spp., while 14 were free of large cestodes (Table 1). The swab-PCR detected 44 samples positive for *Taenia* spp. (sensitivity 89.8%) compared to 41 detected with swab-LAMP (sensitivity 83.7%). For *Mesocestoides* spp., there was one false-negative sample by the swab-PCR (sensitivity 98.0%) compared to seven false negatives using swab-LAMP (sensitivity 86.5%). The specificity of swab-PCR was 85.7%, while that of the swab-LAMP was 78.6% for large cestodes using DNA extraction method A.

**Table 1:** Sensitivity and specificity of the swab-PCR and swab-LAMP in detecting *Taenia* and *Mesocestoides* infections as confirmed by the Sedimentation and Counting Technique (SCT) in 78 foxes using DNA isolation method A^**a**^ and 27 foxes using method B^**a**^

DNA isolation method	Large cestodes	Swab-PCR No. positive/total (95% confidence interval)	Swab-LAMP No. positive/total (95% confidence interval)
A^a^	*Taenia* spp.	44/49^b^ Sensitivity 89.8% (77.7–96.6)	41/49^b^ Sensitivity 83.7% (70.3–92.7%)
	*Mesocestoides* spp.	51/52^b^ Sensitivity 98.0% (89.7–100)	45/52^b^ Sensitivity 86.5% (74.2–94.4)
	Negative	2^e^/14 Specificity 85.7% (57.2–98.2)	3^d^/14 Specificity 78.6% (49.2–95.3)
B^a^	*Taenia* spp.	17/19^c^ Sensitivity 89.5% (66.9–98.7)	17/19^c^ Sensitivity 89.5% (66.9–98.7%)
	*Mesocestoides* spp.	18/19^c^ Sensitivity 94.7% (74.0–99.9)	16/19^c^ Sensitivity 84.2% (60.4–96.6)
	Negative	0/4 Specificity 100% (39.8–100)	0/4 Specificity 100% (39.8–100)

^a^DNA extraction method A: alkaline lysis method and QIAamp kit; method B: alkaline lysis only

^b^37 of these foxes were co-infected with *Taenia* and *Mesocestoides* spp.

^c^15 of these foxes were co-infected with *Taenia* and *Mesocestoides* spp.

^d^one of the three samples is positive by swab-PCR and faecal egg isolation and PCR/ sequencing for *Taenia* spp., while one sample is positive by faecal egg isolation only, and one sample is negative by both swab-PCR and faecal egg-isolation

^e^these two samples were positive for faecal egg isolation and PCR/sequencing for *Taenia* spp.

Nineteen of the 27 samples used for DNA extraction with method B were positive for *Taenia* spp. at necropsy/SCT. The swab-PCR and swab-LAMP detected 17 positive samples (sensitivity 89.5%) using method B. *Mesocestoides* spp. infections were detected in 19 foxes at necropsy/SCT and swab-PCR detected 18/19 (sensitivity 94.7%), while the swab-LAMP detected 16/19 (sensitivity 84.2%). The specificities of the swab-PCR and swab-LAMP were 100% using method B for DNA extraction. There was no significant difference in the sensitivity of the swab-PCR and swab-LAMP to detect *Taenia* spp. and *Mesocestoides* spp. DNA in the various worm burden categories (Table 2). A different number of foxes were used for this study for DNA extraction method A and method B. Nevertheless, the worm burden of the two groups of foxes presented for necropsy and used in DNA extraction methods A and B did not differ significantly (Table S4).

**Table 2:** Sensitivity of swab-PCR and swab-LAMP related to the worm burden of large cestodes (*Taenia* and *Mesocestoides*) determined by the Sediment and Counting Technique (SCT) in naturally infected foxes

DNA isolation method	Genus	Number of worms	Swab-PCR No. positive/total Sensitivity (95% confidence interval)	Swab-LAMP No. positive/total Sensitivity (95% confidence interval)
A^a^	*Taenia* spp.	< 10	31/35 88.5% (73.3–96.8)	30/35 85.7% (69.7–95.2)
		11–20	13/14 92.8% (66.1–99.8)	11/14 78.6% (49.2–95.3)
	*Mesocestoides* spp.	< 10	37/38 97.3% (86.2–99.9)	34/38 89.5% (72.2–97.1)
		11–20	12/12 100% (73.5–100)	9/12 75% (45.8–94.5)
		21–30	1/1	1/1
		> 31	1/1	1/1
B^a^	*Taenia* spp.	< 10	9/11 81.2% (48.2–97.7)	10/11 90.9% (58.7–99.8)
		11–20	8/8 100%(63.1–100)	7/8 87.5% (47.4–99.7)
	*Mesocestoides* spp.	< 10	8/9 88.9%(51.8–99.7)	7/9 77.8% (40.0–97.2%)
		11–20	6/6 100%(54.1–100)	5/6 83.3%(35.9–99.6)
		21–30	3/3	3/3
		> 31	1/1	1/1

^a^DNA extraction method A, alkaline lysis method and QIAamp kit; method B, alkaline lysis only

#### Amplification of *E. multilocularis* and *T. canis* in foxes using peri-anal swabs

Using DNA extraction method A, from the 45 foxes infected with *E*. *multilocularis* at necropsy/SCT, six samples were detected by the swab-PCR corresponding to a sensitivity of 13.3% (95% confidence interval, 5.1– 26.8). LAMP for *E. multilocularis* detected 11 infections, corresponding to a sensitivity of 24.4% (95% CI, 12.8– 39.5). From the 27 foxes used for DNA extraction with method B, we found *E. multilocularis* in 21 animals, from which swab-PCR and LAMP detected only two positives (sensitivity, 9.5%; 95% CI, 1.2–30). The specificity of both methods of DNA extraction for *E. multilocularis* detection was 100%.

In the case of *T. canis*, from the 78 peri-anal swabs used for method A of DNA extraction, worms were identified during necropsy/SCT in 31 foxes. PCR detected six positive infections (sensitivity, 19.4%; 95% CI, 7.5–37.5). Using DNA extraction method B, no positives were detected with PCR compared to 12 infections detected during necropsy/SCT.

## Discussion

The results of the present study have shown the potential of using peri-anal swabs as a sampling method for detecting large cestode DNA. One major challenge in molecular detection of any infective agent in low-income countries is the DNA isolation step which is time-consuming and relies on costly kits (Aula et al. 2021). The results for the application of peri-anal swab in foxes showed that there was no significant difference for both methods of DNA extraction in the detection of large cestodes by swab-PCR and swab-LAMP. DNA extraction method A, a classical method, was developed for taeniid egg isolation in faeces and modified to reduce PCR inhibitors (Štefanić et al. 2004), but it is a laborious method requiring up to 2 h to perform and is not suitable for mass-screening in field surveys. Method B used in this study offers a cost-effective, fast and straightforward alternative DNA extraction protocol (approximately 25 min to perform for ten samples) in a field set-up with only two reagents (NaOH and Tris–HCl), circumventing the need for costly DNA isolation kits. Recently, Mason and Botella (2020) developed a method of purifying DNA from a dipstick without the need for equipment. The dipstick is dipped in an extraction buffer, a wash buffer, and then the eluted DNA is used directly into a PCR or LAMP assay. The publication by Mason and Botella (2020) illustrates the need for cost-effective methods for DNA extraction which can be used in field conditions. In our case, we show that amplifications occurred using the supernatant of NaOH lysis directly used for PCR and LAMP representing an advantage over methods needing DNA purification.

The swab-PCR assay was sensitive in detecting DNA from *Taenia* spp. using methods A and B (89.8% and 89.5%) and *Mesocestoides* spp. (98% and 94.7%). These cestodes have large biomass and metabolic activity with a high egg output, contributing to contamination of the peri-anal region with parasite material. The false-negative samples from the field study could result from prepatent infections. Another probable explanation for the false negatives by both swab-PCR and swab-LAMP in patent infections could be a low DNA concentration below the limit of detection or the presence of inhibitors. Moreover, the grooming of the anal region in foxes (Roddie et al. 2008) could decrease the availability of parasite material and could explain the false negatives.

For LAMP primer design, we targeted the 12S rRNA mitochondrial gene as it has been used previously to design primers for PCR to differentiate cestode species (including *Taenia* spp.) from *E. multilocularis* and *E. granulosus* *s.l*. (Trachsel et al. 2007). Results of the analytical sensitivity showed that the LAMP primers could detect down to 1 pg/ μl which is comparable to a previous study where a LAMP assay for canine taeniids detected between 1 to 10 pg of different *Taenia* spp. (Feng et al. 2017). However, in the study by Feng et al. (2017), species-specific primers were designed making the assay laborious as a sample must be simultaneously tested with five different primer sets. With this in mind, we intended to design a unique primer set for the large cestodes in foxes, this required the inclusion of ambiguities in swab-LAMP primers. The specificity of the LAMP assay showed that the primers could also amplify *T. saginata* and *T. solium* even with mismatches in the forward and backward primers (F3 and B3). In a further study, we recommend that well-validated primers for *T*. *solium and T. saginata* can be used (Nkouawa et al. 2009, 2016) for diagnosis of human taeniosis using the DNA collection method presented here.

In our study, the sensitivity of the swab-LAMP for *Taenia* spp., 83.7% and 89.5% using method A or method B, respectively, was comparable to the swab PCR (89.8% and 89.5%). Previous studies have shown LAMP assay to be more sensitive than PCR as *Bst* polymerase is more tolerant to inhibitors than Taq DNA polymerase used in PCR (Notomi et al. 2000; Kaneko et al. 2007). The results of the swab-LAMP for *Taenia* spp. are comparable to a previous study which showed a sensitivity of 88.4% for human taeniosis from stool samples (Nkouawa et al. 2010).

The sensitivity for detecting *Mesocestoides* spp. in the swab-LAMP was 86.5% using method A and 84.2% using method B. From the seven false-negative reactions by the swab-LAMP with method A for DNA extraction, six of them were positive by the swab-PCR and one negative by both amplification methods. Using method B, the three false negatives by the swab-LAMP were positive by the swab-PCR. The low sensitivity for the swab-LAMP assay for *Mesocestoides* spp. could be explained due to mismatches in the primers for *M*. *litteratus* (Yamasaki et al. 2004) (Notomi et al. 2000). Furthermore, we noted from the spiking experiments that the swab-PCR could detect DNA up to 1–10 pg/μl compared to the swab-LAMP (0.1–1 ng/μl), and this could explain the false negatives in the swab-LAMP.

Using DNA extraction method A, the swab-PCR produced two false-positive samples, and one of these was also positive by the swab-LAMP. The two false-positive samples by swab-PCR were confirmed positive for taeniid faecal egg isolation, and these samples could represent false-negative results of the SCT. Positive PCR results can also be obtained by amplifying DNA from metacestodes that have been ingested by the fox together with the intermediate host or due to contamination during sample collection. Our necropsies were performed in a hood with laminar flow. Although contamination was avoided with foxes wrapped in individual bags, frequent changing of gloves during necropsy, cleaning with sodium hypochlorite and decontaminating the hood with UV light, we cannot exclude this possibility.

Our work is the first proof of concept study for the detection of large cestodes DNA in the peri-anal region. The scotch-tape technique that is based on a similar sample origin but detecting parasite eggs microscopically has been shown to be sensitive in detecting *T. saginata* but not *T. solium* intestinal infections (Sarti et al. 1994; Garcia et al. 1997). It is important to consider the differences in proglottid release of these two *Taenia* spp. for future evaluation of the peri-anal swab diagnostic strategy in humans.

Both the swab-PCR and LAMP were not sensitive in the detection of *E. multilocularis* infections. *Echinococcus* spp. has a low reproduction rate per worm as compared to the large cestodes. Experimental infection with *E. multilocularis* showed that foxes have a higher initial worm establishment but with a shorter life expectancy compared with the racoon dog and dog. Most of the worm burden is lost within 2–3 months of infection, although residual worm burdens can persist for several months (Kapel et al. 2006). Hence, it is likely to sample several foxes with a low worm burden, as shown previously in a study by Hofer et al. (2000) where only 8% of the foxes investigated were infected with more than 10,000 *E. multilocularis* worms harbouring 72% of the total biomass in the fox population.

The detection rate of *T. canis* DNA by PCR was also low. The fox population included had a low intestinal worm burden < 10 worms. Previous studies have detected *T. canis* eggs on the fur of dogs or foxes; however, in most of these cases, no viable eggs have been identified. Hence it has been considered an unlikely risk of infection for dog owners (Deplazes et al. 2011).

## Conclusion

The present study has shown a proof-of-concept that peri-anal swabs are a convenient, non-invasive sampling method, potentially to be used in human or animal studies. We used cotton swabs that have been used traditionally as a cost-effective sample collection method, more recently also in the COVID-19 pandemic. Peri-anal swab collection could be integrated into population studies besides questionnaires and blood sampling for specific testing for cysticercosis and improve the people’s willingness to participate in control programs of this serious zoonosis and subsequently enhance the detection of taeniosis carriers.

## Supplementary Information

Below is the link to the electronic supplementary material.

Supplementary file1 (DOCX 153 KB)

## Data Availability

All data generated or analysed during this study are included in this published article [and its supplementary information files].
