# Supplementary material for: Amplification of cestode DNA from the peri-anal region of naturally infected foxes by PCR and LAMP: proof of concept for a potential sampling strategy for diagnosing human taeniosis
Source: Parasitol Res. 2021 Aug 31;120(10):3451–9. doi: 10.1007/s00436-021-07271-z (PMC8405393; doi:10.1007/s00436-021-07271-z)
Supplement: Supplementary file 1 — Supplementary file1 (DOCX 153 KB) [file 436_2021_7271_MOESM1_ESM.docx]

**Supplementary material**

**Parasitology Research**

**Table S1** PCR primers used for amplification of the flanking region of the GenBank entry for T. polyacantha (DQ408419.1) corresponding to the 12S rRNA

| **Primer name** | **Sequence 5`→3`** | **PCR product size** |
| --- | --- | --- |
| *T. polyacantha* 5`Forward | CATGAGTTGAGTTAAGACCGG | 689bp |
| *T. polyacantha* 5`Reverse | AATAAGCAGCACATAGACTTG |  |
| *T. polyacantha* 3`Forward | TAAGCCAAGTCTATGTGCTGC | 606bp |
| *T. polyacantha* 3`Reverse | AATCATAGTCATAGGCTTATC |  |

**Table S2** Swab-LAMP Primers designed in this study for detecting the common Taenia spp. in the foxes

| **Primer name** | **Primer sequence (5` 3`)** |
| --- | --- |
| 12S rRNA F3 | GATACCCCATTAATRTAYWTTGW |
| 12S rRNA B3 | AYMTTATWAAWGTAACGCATGAA |
| 12S rRNA FIP^a^ | ATCCTTTACACCACACCTT-TAACTAAAATRRTTTGGCAGT |
| 12S rRNA BIP^a^ | ATGTTGGTGTATATCTGRTTTAA- CAGCACATAGACTTGRCTTAA |
| 12S rRNA FL | CCCCTAAWAAGARTCRCTC |

^a^FIP consists of the sense sequence of F2 at the 3′ end and the F1c region at the 5′ end that is complementary to the F1 region. BIP consists of a B2 region at the 3′ end that is complementary to the B2c region and the same sequence as the B1c region at the 5′ end.


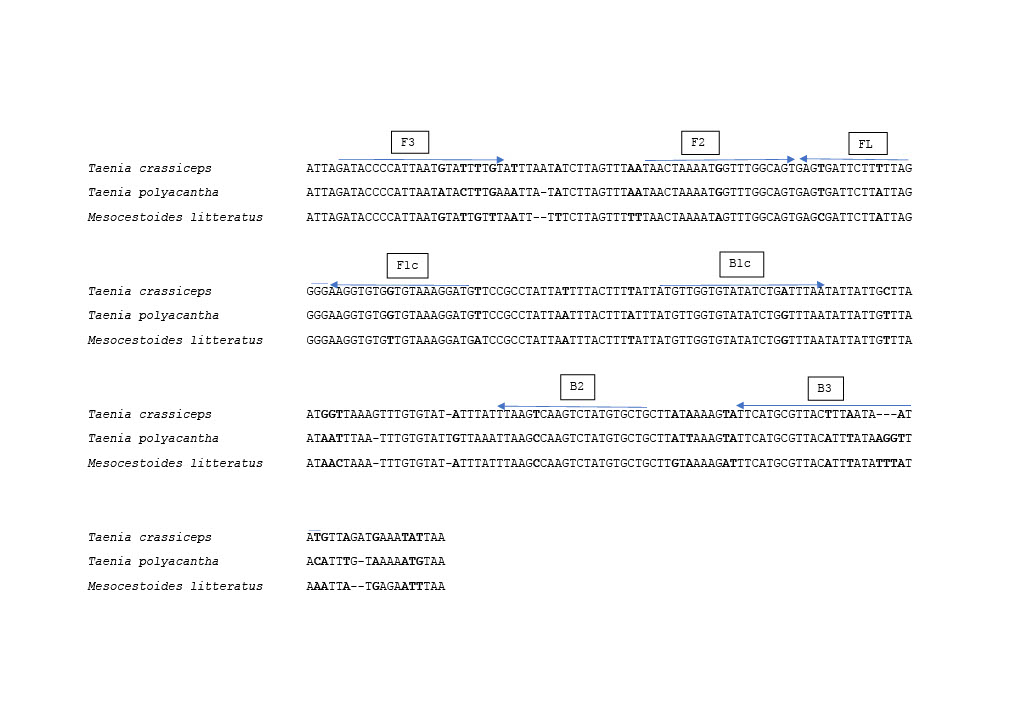


**Fig S1** Nucleotide sequence alignment of the primer region on the 12S rRNA gene for Taenia crassiceps (NC002547), T. polyacantha (MZ414196) and Mesocestoides litteratus (JN088186). The locations of the primer recognition sites are indicated by arrows

**Necropsy and Sediment and Counting Technique Results**

From the 105 foxes presented at the Institute of Parasitology, University of Zurich, 68 foxes were infected with *Taenia* spp*.*, 71 foxes with *Mesocestoides* spp. *Echinococcus multilocularis* were detected in 66 foxes. An uncharacterized cestode species (approximately 2mm) was isolated from 3 foxes. Nematodes, *Toxocara canis* were detected in 43 foxes, while *Uncinaria stenocephala* was detected in 22 foxes. A trematode species*, Alaria* sp. was found in 2 foxes.

**Table S3** Overall Necropsy and Sediment and Counting Technique results of morphologically characterized intestinal helminths in foxes presented by hunters at the Institute of Parasitology (IPZ) from 2020- 2021 (n= 105)

| **Helminth species** | **Number positive** | **Frequency %** |
| --- | --- | --- |
| *Taenia* spp. | 68 | 64.8 |
| *Mesocestoides* spp. | 71 | 67.6 |
| Uncharacterised cestode^a^ | 3 | 2.9 |
| *E. multilocularis* | 66 | 62.9 |
| *T. canis* | 43 | 41.0 |
| *U. stenocephala* | 22 | 21.0 |
| *Alaria* sp. | 2 | 1.9 |

^a^After DNA isolation of the worm using QiAmp DNA Mini kit (Hilden, German), PCR assays were performed targeting the 18S rRNA and 28S rRNA genes as described (Bowles et al. 1992; Littlewood et al. 2000). After sequencing the amplicons from the genes mentioned above the closest homology was 97% with *Parorchite* *zederi* a cestode of penguins (KP893423) and 96% for *Railletina cesticillus*, a cestode of chicken, birds, and other backyard poultry (AY382316). The sequence of the unknown cestode was uploaded in GenBank (MZ420211).


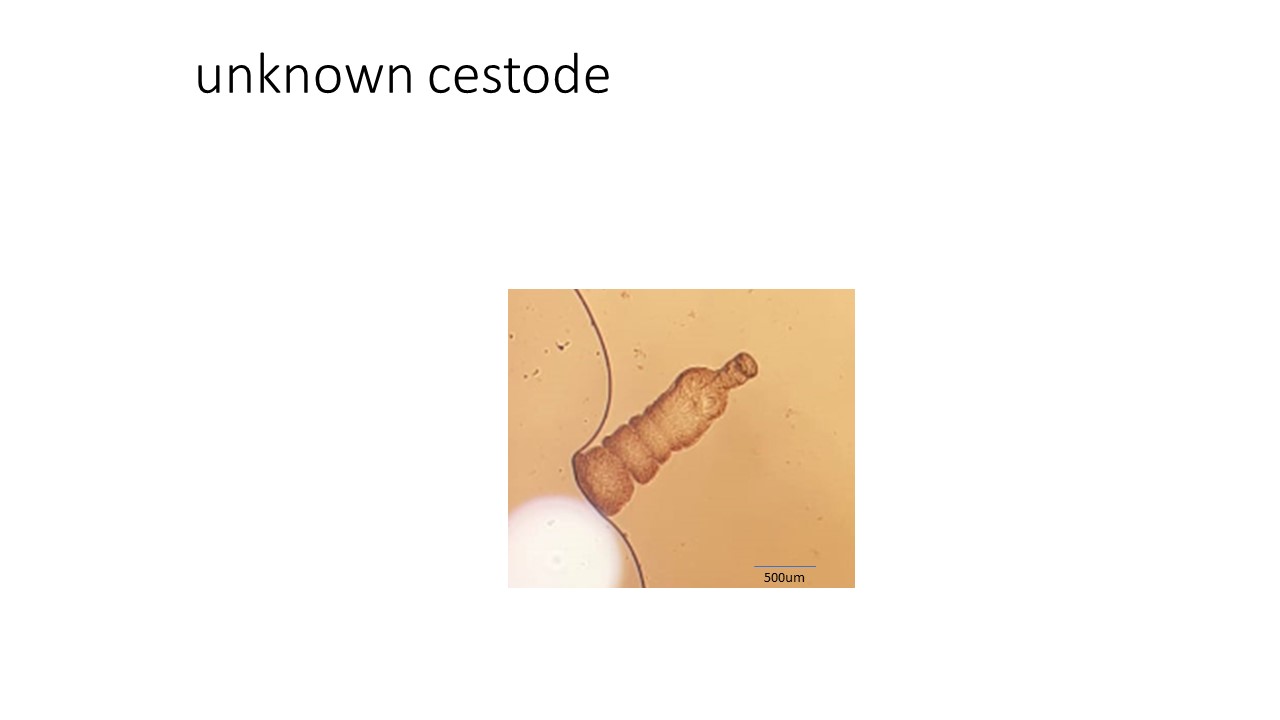


**Fig S2** Image of the uncharacterized cestode (MZ420211) found in the intestinal material in three foxes during the necropsy and SCT.

**Table S4** Infection intensity in the foxes that were used for DNA isolation method A (n= 78) and B (n=27) for the detection of large cestodes (Taenia spp. and Mesocestoides spp.) in this study

| **Genus** | **Number of worms** | **DNA isolation method A**^a^  (Number positive/Total number)  Infection intensity (95% CI) | **DNA isolation method B**^a^  Infection intensity (Number positive/Total number)  Infection intensity (95% CI) |
| --- | --- | --- | --- |
| *Taenia* spp. | < 10 | 35/49  71.4% (56.7-83.4) | 11/19  57.9% (33.5- 79.8) |
|  | 11-20 | 14/49  28.6% (16.6- 43.3) | 8/19  42.1% (20.3- 66.5) |
| *Mesocestoides* spp. | <10 | (38/52)  73.1% (59.0- 84.4) | 9/19  47.3% (24.5- 71.1) |
|  | 11-20 | 12/52  23.1 (12.5- 36.8) | 6/19  31.6% (12.6- 56.6) |
|  | 21-30 | 1/52  1.9% (0.1-10.3) | 3/19  15.8% (3.4-39.6%) |
|  | >31 | 1/52  1.9% (0.1-10.3) | 1/19)  5.2% (0.1- 26.0%) |

^a^DNA extraction method A: alkaline lysis method and QIAamp kit; method B: alkaline lysis only

**Supplementary references**

Bowles J, Blair D, McManus DP (1992) Genetic variants within the genus Echinococcus identified by mitochondrial DNA sequencing. Mol Biochem Parasitol 54(2):165-173 doi:10.1016/0166-6851(92)90109-w

Littlewood DTJ, Curini-Galletti M, Herniou EA (2000) The Interrelationships of Proseriata (Platyhelminthes: Seriata) Tested with Molecules and Morphology. Mol Phylogenet Evol 16(3):449-466 doi:https://doi.org/10.1006/mpev.2000.0802
